# Effect of intraoperative goal-directed fluid therapy on the postoperative brain edema in patients undergoing high-grade glioma resections: a study protocol of randomized control trial

**DOI:** 10.1186/s13063-022-06859-9

**Published:** 2022-11-19

**Authors:** Xiaoyuan Liu, Xingyue Zhang, Yifang Fan, Shu Li, Yuming Peng

**Affiliations:** Department of Anesthesiology, Beijing Tiantan Hospital, Capital Medical University, No.119, Nansihuan Xilu, Fengtai District, Beijing, 100070 People’s Republic of China

## Abstract

**Introduction:**

Brain edema is the most frequent postoperative complication after brain tumor resection, especially in patients with high-grade glioma. However, the effect of SVV-based goal-directed fluid therapy (GDFT) on postoperative brain edema and the prognosis remain unclear.

**Methods and analysis:**

This is a prospective, randomized, double-blinded, parallel-controlled trial aiming to observe whether stroke volume variation (SVV)-based GDFT could improve the postoperative brain edema in patients undergoing supratentorial high-grade gliomas compared with traditional fluid therapy. The patient will be given 3 ml/kg hydroxyethyl starch solution when the SVV is greater than 15% continuously for more than 5 min intraoperatively. The primary outcome will be postoperative cerebral edema volume on brain CT within 24 h.

**Ethics and dissemination:**

This trial has been registered at ClinicalTrials.gov (NCT03323580) and approved by the Ethics Committee of Beijing Tiantan Hospital, Capital Medical University (reference number: KY2017-067-02). The findings will be disseminated in peer-reviewed journals and presented at national or international conferences relevant to the subject fields.

**Trial registration:**

ClinicalTrials.gov NCT03323580 (First posted: October 27, 2017; Last update posted: February 11, 2022).

**Supplementary Information:**

The online version contains supplementary material available at 10.1186/s13063-022-06859-9.

## Strengths and limitations of this study

- This is a randomized, parallel-group, control trial to evaluate whether stroke volume variation (SVV)-guided GDFT could improve the postoperative brain edema in neurosurgical patients with supratentorial malignant gliomas.
- This study adopts SVV-based GDFT, with standardized randomization, endpoint assessment blinding, and experienced investigators in each key procedure of intervention and follow-up.
- One limitation of the study is it is a single-center trial. A future multi-center trial is needed to evaluate the effect of GDFT on the postoperative brain edema in patients under supratentorial malignant glioma resections.
- Certain patients with known high risks for early postoperative complications are excluded for safety concerns.

## Background

Postoperative brain edema induces increased intracranial pressure (ICP), worsened neural function, and increased morbidity and mortality in patients undergoing brain tumor resections [1], which is related to various factors including blood-brain barrier disruption [2], tumor characteristics [3], and systemic hypervolemia.

Intraoperative fluid load is regarded as one of the factors that improve cerebral edema [4] for patients with supratentorial high-grade gliomas. Previous studies revealed sufficient perioperative fluid volume ensures stable circulation and maintains adequate cerebral perfusion pressure. Though strict control of fluid input provides intraoperative brain relaxation, it is also associated with insufficient organ perfusion and other related complications [5], including a risk of decreased cerebral perfusion pressure [6], leading to aggravating cerebral edema and further leading to long-term brain injury [6, 7]. Therefore, how to optimize intraoperative fluid treatment is controversial. Individualized volume monitoring and fluid therapy may be required for patients with different volume statuses.

Goal-directed fluid treatment (GDFT) was proposed by River in 2001 [8] for patients with severe sepsis and septic shock. GDFT provides patients with individualized fluid therapy so as to achieve optimal individual cardiac function and sufficient tissue perfusion. Previous studies showed that intraoperative GDFT remarkably improved prognosis [9–11], shortens the length of hospital stay, and reduces the medical cost [12]. However, there is no research focus on the impact of intraoperative fluid treatment on postoperative brain edema, especially in patients with high-grade gliomas.

Stroke volume variation (SVV) is a way to evaluate the volume status during surgery. It was measured by the pulse contour method and predicts the response to fluid administration instantly and continuously. It has been commonly used in various settings. GDFT was applied in thoroesophagectomy guided by SVV of less than 8% and was demonstrated to reduce the major morbidity and mortality compared with the control group, in which systolic blood pressure was maintained greater than 90mmHg [13]. A meta-analysis of 14 studies indicated that GDFT reduced postoperative morbidity and length of stay in the ICU [14]. Recently, SVV had been reported to be sensitive in predicting fluid responsiveness in neurosurgery. Xia et al. [11] studied the effects of different kinds of fluids applied by GDFT in patients undergoing supratentorial tumor resection and found satisfactory brain relaxation was both achieved, whereas no difference in cerebral ischemia, lactic acid production, or increased glucose intake between groups was found. Luo et al. involved patients undergoing brain tumor surgery and found that intraoperative use of GDFT reduced the length of stay in the ICU of high-risk neurosurgical patients [15]. However, the researchers did not set a specific fluid regimen for the control group but based on the experience of the anesthesiologist. Besides, the study population included patients mostly with brain vascular diseases, resulting in a relatively insufficient sample size of brain tumors.

The cut-off value of SVV is generally set at 9–15% [16, 17]. Wu et al. [18] investigated the effects of GDFT with different SVV target values (SVV≤10% or SVV≤18%) in patients undergoing supratentorial cranial tumor surgery and found the incidence of postoperative neurological events was lower in patients with SVV≤10% group. Luo et al. found [15] maintaining SVV<15% not only ensured stable circulation and adequate tissue perfusion but also limited the amount of fluid input on the basis of satisfying tissue perfusion. However, there is still no study focusing on the effects of SVV-guiding GDFT on postoperative brain edema in neurosurgical patients.

Therefore, the purpose of the study is primarily to explore whether SVV-guiding GDFT optimizes intraoperative fluid treatment concerning reducing postoperative cerebral edema volume in patients with malignant supratentorial gliomas. We will conduct a randomized controlled trial to test the hypothesis that SVV-guiding GDFT could reduce postoperative cerebral edema volume compared with traditional fluid treatment.

## Methods

### Study design

**Fig. 1:**
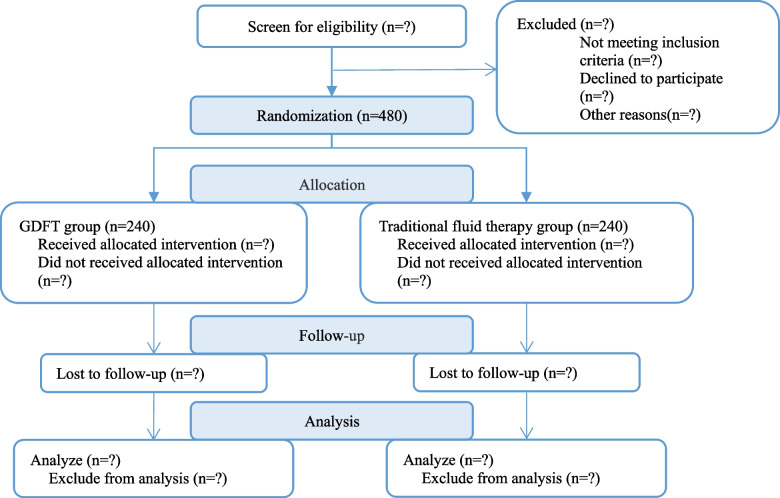
Flow chart of the study. GDFT goal-directed fluid therapy

This is a prospective, randomized, endpoint assessor-blinded, parallel-controlled trial. Supratentorial malignant tumor patients will be recruited from Beijing Tiantan Hospital, Capital Medical University, from 2017 to 2022. This trial was approved by the Medical Ethics Committee of Beijing Tiantan Hospital, Capital Medical University (reference number: KY2017-067-02), on 27 November 2017 and registered in www.clincaltrials.gov (NCT03323580). Preoperative interviews will be conducted by specially trained research assistants who inform patients of the study objectives, risks, and benefits and obtain written informed consent from his/her legal representatives. Figure 1 shows the flow chart of the study.

### Subjects

#### Inclusion criteria

Patients scheduled to undergo elective supratentorial tumor resection with preoperative brain image indicating high-grade glioma (HGG) will be recruited for screening eligibility 1 day before surgery. The inclusion criteria include age between 18 and 65 years and American Society of Anesthesiologists (ASA) physical status I to III. HGG will be verified by postoperative histology of the World Health Organization (WHO) as grade III or IV tumors [19]. Informed consent will be signed by the patient or his/her legal representative.

#### Exclusion criteria

Patients will be excluded from the study if the patients are with recurrent carcinoma, tumor in the brain ventricular, New York Heart Association Functional Classification (NYHA) functional class of II–IV or if their cardiac ejection fraction is < 20%, chronic obstructive pulmonary disease, renal insufficiency, creatinine clearance rate < 30 ml/kg, extensive peripheral arterial occlusive disease, coagulopathy, surgery in the prone position, body mass index <18.5 kg m^−2^ or >30.0 kg m^−2^, and awake craniotomy.

### Randomization and blinding

Block randomization (block size of 6) will be applied via a computer-produced randomized table. Patients will be randomized within 24 h before surgery. An independent research assistant will pack the allocation sequence with opaque, sealed, and stapled envelopes and distribute it to the responsible anesthesiologist.

The endpoint assessors (neuroradiologists who will analyze the postoperative CT scan and calculate the brain edema volume) will be all blinded to the grouping until the completion of the study analysis unless specific circumstances, including the occurrence of a serious adverse event. The enrolled patients and the legal representatives will also be blinded to the interventional treatment.

### Intervention

Patients will be randomly assigned to the GDFT group and the traditional treatment group. In the GDFT group, the A-line will be connected to the EV1000A (Edwards Lifesciences, Irvine, CA, USA) to obtain the SVV, stroke volume index (SVI), and cardiac index (CI). The Vigileo/Flotrac system will analyze the pressure waveform 100 times per second over 20s, capturing 2000 data points for analysis and performing calculations by using data obtained in the most recent 20s. Each parameter will be recorded as a number once a minute. After levering, flushing, and baseline calculation, the fluid infusion will be performed according to the following protocol until the end of the surgery.

**Fig. 2:**
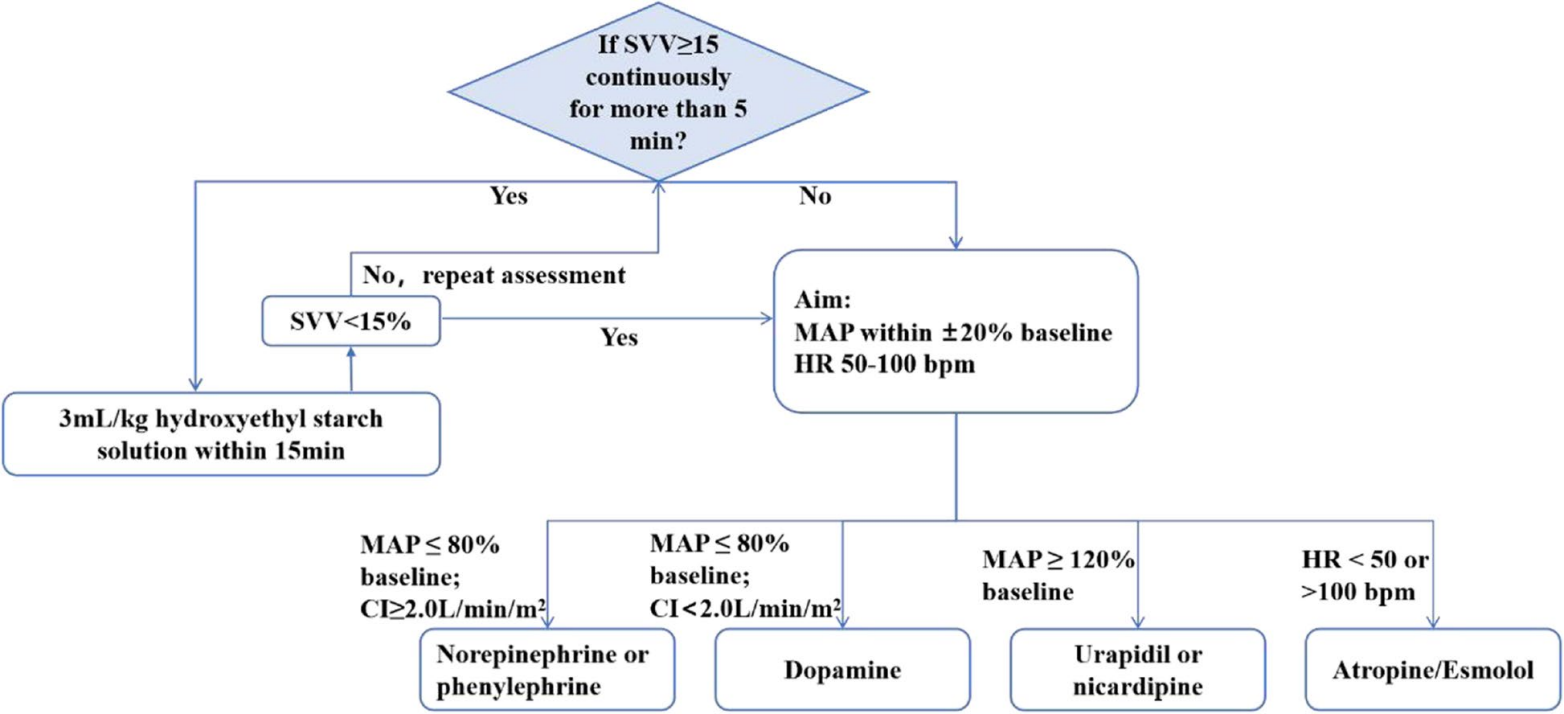
GDFT protocol. SVV stroke volume variation, MAP mean arterial pressure, HR heart rate, Hb hemoglobin, GDFT goal-directed fluid therapy

Two periphery intravenous accesses will be established to ensure fluid administration. The attending anesthesiologist will evaluate the parameters every 15 min. If SVV ≥ 15% occurs continuously for at least 5 times during 15 min, a 3-ml/kg bolus of hydroxyethyl starch will be infused within the next 15 min through one intravenous access. When the number of the bolus is up to 5, hydroxyethyl starch will be replaced by acetate Ringer’s solution for further bolus infusion. If SVV is less than 15% and the MAP is lowered by more than 20% of the baseline value, norepinephrine or phenylephrine will be used to raise blood pressure if CI (cardiac output index)≥2.0 l/min/m^2^ in the GDFT group. If CI < 2.0 l/min/m^2^, dopamine will be given. In the traditional fluid therapy group, norepinephrine, phenylephrine, or dopamine will be given by experience. When MAP increases by more than 20% from the baseline value, urapidil or nicardipine will be administered. When HR is less than 50 bpm, atropine 0.2 to 0.5mg will be given, and when HR is higher than 100bpm, esmolol will be infused 10 to 20mg in bolus or continuously (see Fig. 2). Acetate Ringer’s solution will be infused at a fixed rate of 3 ml/kg h for basic fluid maintenance through another periphery intravenous access during the whole process.

In the traditional fluid therapy group, the fluid volume is given according to the experience of the anesthesiologist. The intraoperative target value of MAP and HR was maintained and the protocol of cardiovascular agent administration is as the same as the GDFT group.

If bleeding in the GDFT group exceeds 3ml/kg/min for more than 20 min, or estimated blood loss exceeds 20% of total blood volume and circulation is difficult to maintain according to the GDFT protocol, the attending anesthesiologist will decide whether to violate the GDFT protocol and take emergent measures for safety.

### Anesthesia induction and management

Routine monitoring will include non-invasive blood pressure (NBP), electrocardiography (ECG), pulse oxygen saturation (SPO_2_), end-tidal carbon dioxide partial pressure (PetCO_2_), bispectral index (BIS), body temperature, and urine output. The radial artery is punctured to monitor SVV, CI, SVI, and other parameters. After the two intravenous accesses are established using 18-G catheters, the participants will get about 300ml of acetate Ringer’s solution before induction. A 22-G arterial line (A-line) will be inserted at the radial arterial immediately after induction. Preoperative midazolam (0.02–0.05 mg/kg) will be given intravenously. Anesthesia will be induced with sufentanil, rocuronium or cisatracurium, and propofol or etomidate and maintained with total intravenous anesthesia by propofol and remifentanil. The dose of propofol will be adjusted to maintain BIS within 40 to 60. Other sedatives (e.g., dexmedetomidine, inhaled anesthetics) will not be administered to maintain anesthesia. Analgesia will be supplemented with sufentanil to attenuate the potential pain stimuli, including skull pin fixation, scalp incision, and dura suture. In addition, the infusion rate of remifentanil will also be adjusted according to the strength of the pain stimulus. Intravenous rocuronium or cisatracurium will be administered as needed.

After tracheal intubation, mechanical ventilation will be performed with the following parameters: tidal volume of 8 to 10 ml/kg, respiratory rate of 10 to 15/min to maintain PetCO_2_ 30–35 mmHg, 40–60% inspired oxygen fraction, and fresh gas at a flow rate of 1 to 2 l/min with positive end-expiratory pressure as 0 to 5 cmH_2_O. Before incision, additional scalp local infiltration with 0.5% ropivacaine will be given. All patients will receive 250 ml of 20% mannitol infusion for 20 min at the start of the scalp incision. No other dehydration drugs will be given during the operation. In addition, the perioperative use of hypotonic sugar-containing fluids will be avoided to prevent aggravating cerebral edema. For all of the participants, the MAP will be maintained higher than 80% of the baseline value and HR will be maintained within 50 to 100 bpm throughout the surgery. Hemoglobin level will be maintained at more than 7 g/dl.

### Data collection and measurement

All patients will receive MRI to assess the tumor size, location, vascularization, and brain edema before surgery. The degree of preoperative brain edema will be assessed according to the Steinhoff classification: 0, no signs of edema; I, mild edema, limited to 2 cm; II, moderate edema, > 2 cm but limited to the ipsilateral hemisphere; and III, severe edema, extending to the contralateral hemisphere [20]. Demographic information and past history of patients were recorded during preoperative screening. Comorbidity was ranked according to the Charlson Comorbidity Index that categorizes comorbidity based on the International Classification of Diseases diagnosis codes. The functional status will be assessed using Karnofsky Performance Status (KPS) at admission and 30 days after surgery. The physiological parameters, the total doses of anesthetics, will be recorded by anesthesiologists through a designed data collection table.

All patients will receive a plain CT scan within 24 h, postoperatively. Plain CT will be performed with a 16-row multidetector scanner (Discovery CT 750HD, GE Healthcare, Milwaukee, USA), a 64-row multidetector scanner (LightSpeed VCT, GE Healthcare, Milwaukee, USA), and a 256-row multidetector scanner (Revolution CT, GE Healthcare, Milwaukee, USA). The scanning parameters will be as follows: tube voltage, 120kV; tube current, 300mA; field of view, 23cm×23cm; and matrix, 512×512. Imaging data will be measured by using a picture archiving and communication system (NEUSOFT PACS/RIS v2.1, Shenyang, China). The maximum diameters of the tumor will be measured on axial, coronal, and sagittal images and defined as *x*, *y*, and *z*. The volume will be calculated as the following formula: *V* = 4/3*π* × *x*/2 × *y*/2 × *z*/2 [21].

Postoperative complications (defined in Supplementary Table 1) and all-cause mortality will also be recorded. Long-term follow-up will be performed through telephone or a remote video interview to collect information.

### Outcomes

**Fig. 3:**
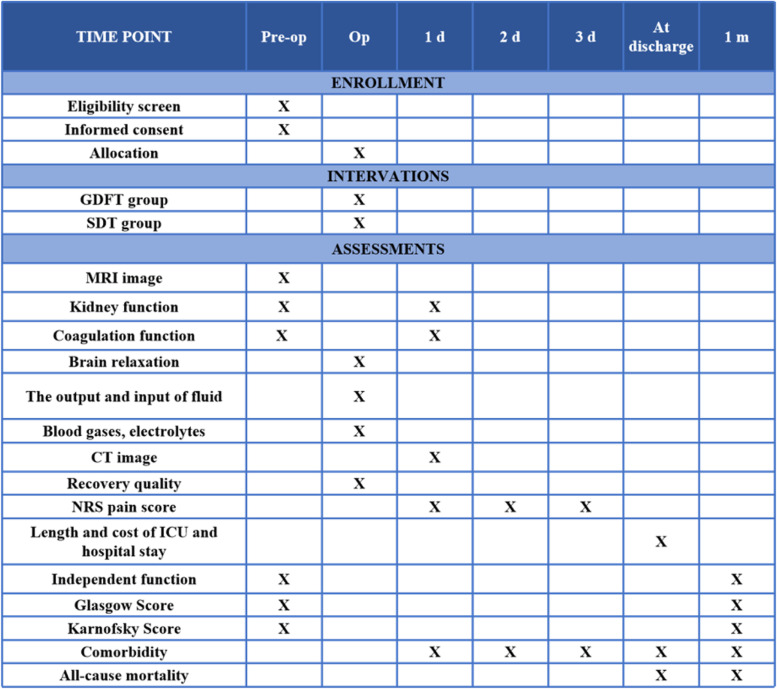
Data collection at each time point. Pre-op preoperative, Op operative, GDFT goal-directed fluid therapy, SDT standard treatment, MRI magnetic resonance imaging, CT computerized tomography, NRS Numeric Rating Scale

This study aims to demonstrate whether SVV-guiding GDFT could reduce the postoperative brain edema volume in patients with high-grade supratentorial gliomas compared with traditional fluid therapy. The assessment of primary and secondary outcomes will be performed by a neuroradiologist and trained researchers blinded to the group allocation. Figure 3 shows the data collection at each time point.

#### Primary outcome

The primary outcome is brain edema volume within 24 h postoperatively by CT examination. Image evaluation and analysis will be performed by two independent neuroradiologists. They are specifically trained to detect cerebral edema on CT based on the following methods for 3 months before evaluation. Both of them will be blinded to the clinical data.

Postoperative brain edema is defined as edema surrounding the surgical resection cavity that has a low density on CT images. Image evaluators manually delineate a region of interest (ROI) and the operative cavity on each slice. The area (*S*_edema+cavity_ and *S*_cavity_) will be calculated automatically by the PACS (picture archiving and communication system, NEUSOFT PACS/RIS v2.1, Shenyang, China). The total volume will be acquired by multiplying the area and slice thickness. We will obtain the total volume of the abnormal density or signal (*V*_edema+cavity_) and the volume of the cavity (*V*_cavity_). The volume of edema (*V*_edema_) will be calculated by the following formula: *V*_*edema*_ = *V*_*edema* + *cavity*_ − *V*_*cavity*_.

#### Secondary outcomes

1. Brain relaxation will be evaluated upon opening the dura by neurosurgeons. Brain relaxation is divided into four grades: completely relaxed, satisfactorily relaxed, firm brain, and bulging brain [22, 23].
2. The blood gas, electrolytes, hematocrit, lactic acid, and glucose at the beginning of fluid management and the closure of the skin incisions.
3. The renal function (urea, creatinine, estimated glomerular filtration rate) and coagulation function (fibrinogen degradation products, D dimer, prothrombin time, international normalized ratio, activated partial thromboplastin time, fibrinogen) on postoperatively 1 day.
4. Recovery quality will be evaluated by the quality of recovery-15 score (QoR-15) in five aspects (physical comfort, physical independence, psychological support, emotion, and pain) after patients recover from general anesthesia within the first 24 h.
5. Incidence of severe pain (numerical rating scale≥5) within 24 h and at the 1, 3, 5, and 7 days after surgery will be assessed.
6. Length of ICU and hospital stay and total hospitalization cost will be recorded.

### Data encoding and storage

The data of all subjects will be coded and this coding will not be retraceable to the individual patient. The key to this coding will be safeguarded by the principal investigator. Only the principal investigators and statistical participants will have access to the source data. In addition, the study monitor and ethical office will have access to the source data.

All collected data will be kept strictly confidential for research purposes only. The paper case report form and electronic data collection form will be used at the same time. Files containing information from the participants will be stored in a locked filing cabinet. The data will be stored in a research computer and backup will be performed on a weekly basis on another external hard drive. Documents will be stored safely in confidential conditions.

### Data monitoring committee

The project will be monitored by the Data Monitoring Committee (DMC) composed of specialists in anesthesiology, neurosurgery, ethics, neuroradiologist, statistics, and methodology. The DMC will conduct audits through regular interviews or telephone. The DMC is responsible for terminating the research in case of a severe adverse event.

### Sample size calculation and statistical plan

We used the PASS 2011 software (NCSS LLC, USA) for Windows to calculate the sample size. According to the pilot study we conducted, the sample size of 450 will find the difference in postoperative brain edema volume of 4 cm^3^ with a standard deviation of 18 and 16 in the two groups. Considering the drop-out rate of 5%, we calculated the sample size of 480. In addition, we also estimated the sample size based on previous studies reporting the incidence of brain edema varying from 6.8 to 50% after malignant brain tumor resection. The sample size of 480 patients (240 for each group) will be sufficient to detect the difference of 13% at a two-tailed significant level of 0.05 and a power of 80% using the Student’s *t*-test, with a drop-out rate of 5%. Our medical center completed about 800 high-grade glioma resection surgeries each year, which could ensure enough participants.

Descriptive statistics will be reported as means with standard deviation and medians with inter-quartile range (IQR) for normally distributed data and skewed continuous data, respectively, and counts (percentage) for categorical data. Normally distributed continuous variables will be compared with Student’s *t*-test, while skewed variables will be compared using the Mann-Whitney *U* test. The categorical variables will be compared with *X*^2^ analysis or Fisher’s exact test. The repeated measurement data will be analyzed by repeated measurements of variance analysis with Bonferroni correction. The primary outcome, brain edema volume, will be compared between groups using the Student’s *t*-test on intention to treat and per protocol, and the conclusion will be drawn according to the intention-to-treat analysis.

The intention-to-treat analysis will depend on the allocated population while the per-protocol analysis will depend on the actual fluid therapy the population receives. Furthermore, subgroup analysis is required in this study, and patients will be analyzed according to preoperative ASA physical status, KPS score, and WHO classification given by postoperative pathology. In addition, missing data will be imputed using inverse probability weighting and the worst-case imputation scenarios for sensitivity analysis will be performed for missing values of primary outcomes. SPSS 16.0 for windows will be used for all statistical analyses. The statistical significance will be declared at a type I error of 0.05.

### Ancillary and post-trial care

The traditional fluid therapy and GDFT adopted in this study are conventional and currently widely recommended optimized liquid therapy, respectively. All remaining treatments will be performed according to clinical practice and no additional ancillary care will be required. Therefore, there is no expected compensation for participation in the trial. However, as post-trial care, participants could contact the study team at any time after the intervention.

### Reporting adverse events

Adverse events include intraoperative sudden cough and body movement, acute brain bulging, postoperative renal failure, respiratory and circulatory arrest, acute myocardial infarction, acute pulmonary embolism, arrhythmia, and massive cerebrovascular infarction. All adverse events associated with the trial will be recorded and closely monitored until it has been proved that intraoperative fluid therapy is not the cause of the event. The principal investigator is responsible for reporting all adverse events. Once an adverse event occurs, it should be immediately reported to the research department and informed to the principal investigator to determine the severity of adverse events. All adverse events associated with this study will be recorded and reported to the Ethics Committee within 24 h.

## Discussion

This is a prospective, randomized, double-blind, parallel-controlled clinical trial aiming to observe the effects of intraoperative GDFT on the postoperative brain edema volume in neurosurgical patients with high-grade supratentorial gliomas.

In this study, we choose acetate Ringer’s solution as a crystalloid fluid to supplement the preoperative fluid loss and intraoperative physiological requirements. Acetate Ringer’s solution has the advantage of being close to human physiology in composition and osmotic pressure, which will avoid perchloric acidosis caused by a large amount of normal saline input, the low osmotic pressure, and subsequent cerebral edema if sodium lactate Ringer’s solution [24] is administered. Moreover, we uniformly give about 300ml of crystalloid solution before induction of anesthesia to supply part of the preoperative loss. Combined with previously related studies, the basic intraoperative infusion rate will be mostly maintained at 1–4 ml/kg/h [11, 15, 18].

Hydroxyethyl starch is currently the colloid most commonly used [25]. Although studies [26] had pointed out that within the clinically recommended dose range (<33ml/kg) [27], the coagulation function is not affected [26], but for patients undergoing brain surgery, intraoperative hemostasis is based on electrocoagulation, which is different from the normal coagulation in the surgical field. Considering the potential impact of a large amount of hydroxyethyl starch on the coagulation function, we have set the upper limit colloid dose close to the clinically commonly used dose of 15ml/kg, which is the amount of 5 boluses for prudential reasons. Furthermore, if the conditions are met and bolus pulse therapy is needed, 3 ml/kg crystalloid solution is used instead of hydroxyethyl starch.

Cerebral edema is the primary outcome of this study. Plain CT is not the optimal image examination in evaluating cerebral edema after craniocerebral surgery, compared to MRI. However, considering that the degree of early postoperative cerebral edema is more closely related to intraoperative fluid management, the study stipulates that imaging examinations should be performed as soon as possible within 24 h after surgery to evaluate cerebral edema. MRI examination takes a long time and the image effect requires the cooperation of patients, whereas patients with high-grade gliomas often present poor consciousness and low cooperation in the early postoperative period. In addition, it is often difficult for patients to enter the MRI machine due to the head dressings and tumor cavity drainage devices. Early MRI examination within 24 h after surgery is not clinically feasible. In neurosurgery, CT examination is usually performed 6 h postoperatively. CT has the advantages of short examination time, low requirements for patient cooperation, and lower price, so it is more suitable for post-cranial surgery examinations. Therefore, we choose to use CT to measure postoperative cerebral edema.

## Summary

In summary, we need to carry out a randomized controlled trial to determine the effect of intraoperative GDFT on postoperative brain edema. If intraoperative GDFT improves postoperative brain edema and reduces the incidence of perioperative complications in patients with malignant supratentorial glioma in neurosurgery, SVV-guiding GDFT would be a good choice to decrease the brain edema for patients undergoing malignant supratentorial glioma resection.

## Supplementary Information

**Additional file 1: Supplementary Table 1.** Postoperative complications.

## Data Availability

Following the publication of research results, a completely anonymous set of data can be made available upon reasonable scientific request and with ethical approval. Appropriate credit or co-authorship must be awarded to the author of the study, depending on the extent of data use and planned research. The CRF samples used in this study are available upon reasonable scientific request.
