# Supplementary material for: Effect of intraoperative goal-directed fluid therapy on the postoperative brain edema in patients undergoing high-grade glioma resections: a study protocol of randomized control trial
Source: Trials. 2022 Nov 19;23:950. doi: 10.1186/s13063-022-06859-9 (PMC9675213; doi:10.1186/s13063-022-06859-9)
Supplement: Supplementary file 1 — Additional file 1: Supplementary Table 1. Postoperative complications. [file 13063_2022_6859_MOESM1_ESM.docx]

Supplementary table 1. Postoperative complications

| Postoperative complications | Diagnosis |
| --- | --- |
| Myocardial infarction | Confirmed by elevated troponin or creatine kinase with symptoms, electrocardiogram, coronary angiography, and autopsy. |
| Cardiac arrest | Confirmed by electrocardiogram. |
| Pulmonary embolism | Confirmed by CTPA, autopsy |
| Stroke | New neurological deficits lasting longer than 24 hours (assessed by a neurologist) and reported by CT or MRI as intracranial ischemia. |
| Cerebral hematoma or edema | Intracranial hematoma or edema reported on CT or MRI with altered consciousness. |
| Hydrocephalus | Intracranial hematoma or edema reported on CT or MRI with altered consciousness. |
| Second craniotomy | Intracranial hematoma or edema reported on CT or MRI with altered consciousness. |
| Septic shock | Sofa score ≥2 |
| Surgical SiteInfection | Inflammation, purulent secretion, positive secretion culture. |
| Other complications |  |

CTP: computer tomography pulmonary angiography; CT: computerized tomography; MRI: magnetic resonance imaging; Sofa: Sequential organ failure asses.
